# Forest degradation and biomass loss along the Chocó region of Colombia

**DOI:** 10.1186/s13021-019-0117-9

**Published:** 2019-03-23

**Authors:** Victoria Meyer, Sassan Saatchi, António Ferraz, Liang Xu, Alvaro Duque, Mariano García, Jérôme Chave

**Affiliations:** 1 Jet Propulsion Laboratory, California Institute of Technology, Pasadena, CA USA; 2 Institute of the Environment and Sustainability, University of California, Los Angeles, CA 90095 USA; 3 Departamento de Ciencias Forestales, Universidad Nacional de Colombia, Calle 59A No. 63-20, Medellín, Colombia; 4 Environmental Remote Sensing Research Group, Department of Geology, Geography and Environment, University of Alcalá, Alcalá de Henares, Spain; 5 Laboratoire Evolution et Diversité Biologique, UMR 5174, CNRS Université Paul Sabatier, Toulouse, France

**Keywords:** Lidar, Biomass, Forest height, Tropical forest, Remote sensing, Random forest, Wood density, Forest degradation, REDD+

## Abstract

**Background:**

Wet tropical forests of Chocó, along the Pacific Coast of Colombia, are known for their high plant diversity and endemic species. With increasing pressure of degradation and deforestation, these forests have been prioritized for conservation and carbon offset through Reducing Emissions from Deforestation and forest Degradation (REDD+) mechanisms. We provide the first regional assessment of forest structure and aboveground biomass using measurements from a combination of ground tree inventories and airborne Light Detection and Ranging (Lidar). More than 80,000 ha of lidar samples were collected based on a stratified random sampling to provide a regionally unbiased quantification of forest structure of Chocó across gradients of vegetation structure, disturbance and elevation. We developed a model to convert measurements of vertical structure of forests into aboveground biomass (AGB) for terra firme, wetlands, and mangrove forests. We used the Random Forest machine learning model and a formal uncertainty analysis to map forest height and AGB at 1-ha spatial resolution for the entire pacific coastal region using spaceborne data, extending from the coast to higher elevation of Andean forests.

**Results:**

Upland Chocó forests have a mean canopy height of 21.8 m and AGB of 233.0 Mg/ha, while wetland forests are characterized by a lower height and AGB (13.5 m and 117.5 Mg/a). Mangroves have a lower mean height than upland forests (16.5 m), but have a similar AGB as upland forests (229.9 Mg/ha) due to their high wood density. Within the terra firme forest class, intact forests have the highest AGB (244.3 ± 34.8 Mg/ha) followed by degraded and secondary forests with 212.57 ± 62.40 Mg/ha of biomass. Forest degradation varies in biomass loss from small-scale selective logging and firewood harvesting to large-scale tree removals for gold mining, settlements, and illegal logging. Our findings suggest that the forest degradation has already caused the loss of more than 115 million tons of dry biomass, or 58 million tons of carbon.

**Conclusions:**

Our assessment of carbon stocks and forest degradation can be used as a reference for reporting on the state of the Chocó forests to REDD+ projects and to encourage restoration efforts through conservation and climate mitigation policies.

**Electronic supplementary material:**

The online version of this article (10.1186/s13021-019-0117-9) contains supplementary material, which is available to authorized users.

## Background

Tropical forests have a prime role in carbon balance, biodiversity and society. They contribute significantly to global terrestrial carbon stocks, host more than half of Earth’s species [1], and millions of people depend on forests for food, timber, and other economic and ecosystem services.

Yet tropical forests remain understudied and their carbon stocks are poorly estimated, notably due to the lack of available data, compared to temperate regions [2]. Improving carbon stocks estimations in the tropics, in particular in poorly known regions, would help understanding the global carbon budget better and would enable these regions to participate in projects such as Reducing Emissions from Deforestation and forest Degradation (REDD+). These programs require regions or countries to report on the state of their forest to be able to qualify for carbon credits. In this paper, we focus on one of these regions: the Chocó region.

The lowland Pacific coast region of Colombia, Chocó, has been understudied due to its remote location and history. Famous botanist Alwyn Gentry conducted research in that region during the 1980s [3, 4], revealing that the Pacific coast region of Colombia has an outstanding biodiversity [5, 6] and is a major biodiversity hotpot [6]. There, about 20% of the plant species are endemic, in part because the Pacific Ocean and the western Andean mountain range encircling the region [7] have historically acted as a bridge between Central America and Amazonia. However, the region is under threat of deforestation and degradation. Annual deforestation rate in Colombia has been estimated to 219,973 ha per year [8], of which 6.1% (13,474 ha) comes from the Chocó region, which has suffered from significant ecosystem degradation [9]. In surrounding regions such as the Northwest Pacific Coast of Ecuador and Amazonian Colombia, extensive studies report high rates of degradation, mostly related to commercial logging and land conversion towards agriculture [10, 11].

This study reports on the result of a project, called BioREDD, that was implemented in 2013 to estimate carbon emissions in the Pacific coastal region of Colombia [12]. Eight areas of three subregions were selected along the Pacific coast of Colombia for developing REDD+ projects. These areas cover a variety of ecosystems, from coastal mangroves (“Manglar”) and wetlands (“Guandal”) to terra firme forests on rugged landscapes (“Colinas”). The project was implemented in the territories of Afro-colombian and indigenous communities who have agreed to the REDD+ project development, with the expectation of generating revenues in exchange for their conservation efforts. In these areas, forests have been degraded and are under threat of further degradation and deforestation, due to timber extraction for fuel and development needs, illegal logging, gold mining, and conversion of forests to agriculture and livestock.

Virtually no environmental data was available prior to this study because the region had been considered too remote and difficult to access. We designed a strategy combining airborne lidar data and field plots to create a representative sample of the forest structure and biomass of the region. We complemented these data with space borne data to predict forest height and aboveground biomass (AGB) across the region. Specifically, we ask how limited ground and airborne data can be used to infer a region-scale map of aboveground biomass, together with its uncertainty. We then discuss the implications of our findings for a REDD+ strategy in the Chocó region.

## Methods

**Fig. 1:**
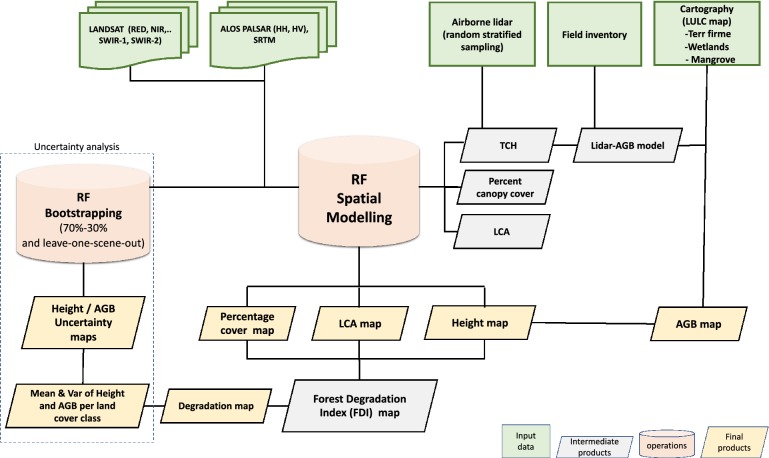
Flowchart showing the steps leading to the production of an AGB map. Data inputs are shown in green, intermediate products are shown in grey, while operations are shown in pink and final products in yellow. Operations and products related to the uncertainty analysis are shown in a dashed box. *AGB* aboveground biomass, *TCH* mean top canopy height, *LCA* large trees canopy area

The methodology (Fig. 1) has four distinct and integrated components including: (1) ground and airborne lidar sampling based on a certified carbon standard methodology, (2) development of unbiased lidar biomass models to convert the lidar height metric to AGB, (3) use of a machine learning algorithm to develop a wall-to-wall map of height and AGB of forests of Chocó over the entire Pacific coastal region of Colombia, and (4) assessment of variations of forest structure and biomass density across the Chocó region by quantifying the uncertainty of estimates.

### Study area

**Fig. 2:**
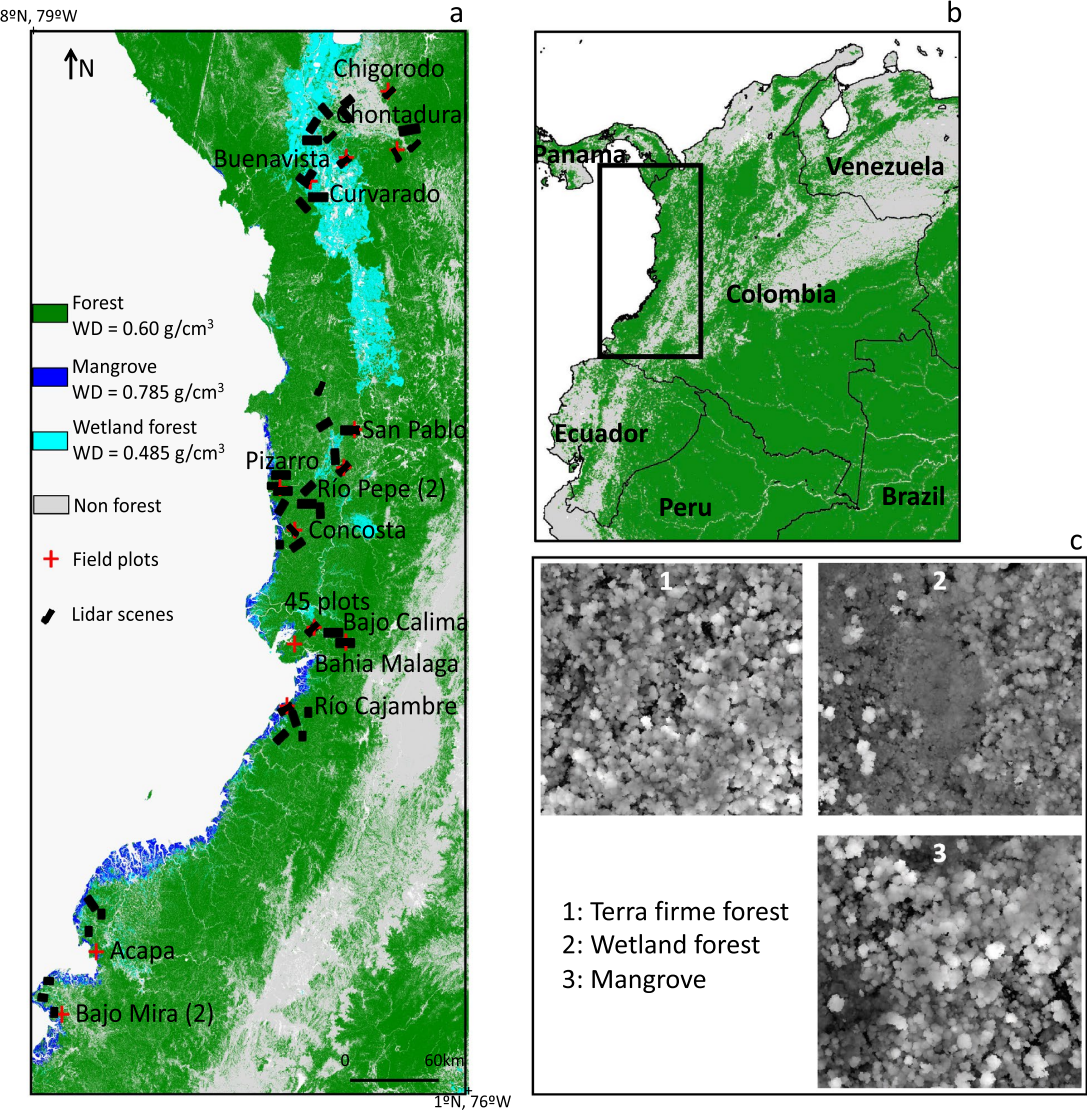
Location of lidar scenes and ground plots over forest, wetland forest and mangrove (**a**). Lidar scenes are not to scale for visualization purposes. Location of study area in South America (**b**). Examples of lidar canopy height models of terra firme forest, wetland forest and mangrove (**c**)

The mapped area is located over a 7° by 3° region, in the Northwest of South America. The study area covers more than 14.5 million ha, of which almost 10 million ha are forested (Fig. 2). Colombia defines a forest as an area greater than 1 ha, with a minimum tree cover of 30% and a minimum tree height of 5 m [13]. The vast majority of the forest area (90.8%) is covered by ‘terra firme’ forest on dry soils extending to higher elevation of Andes including montane forests over elevations greater than 500 m above sea level (asl). Wetland forests (Guandal) cover 7.8% of the forested area in coastal zone at elevations below approximately 50 m from the sea level and mainly in the northern part of the region, along the Atrato River. Mangroves cover an additional 1.4% of the area along the brackish waters of the coast. These vegetation classes are mapped at 30 m spatial resolution based on a land use land cover (LULC) map that was validated during the BioREDD project, for which the airborne lidar data were used to train Landsat imagery to perform a supervised classification (see details in Additional file 1: SI.1; [12]). The terra firme forest class was divided into two subclasses, based on observations in the field and analysis of the lidar data: intact forest (characterized by > 75% tree cover and height > 5 m; 63% of the area) and degraded and secondary forests (30–75% of tree cover; 37% of the area), from multi-temporal Landsat analysis performed to establish the reference emission levels for the BioREDD projects (Additional file 1: Fig. S1).

### Lidar and ground data sampling methodology

We combined four data layers representing climate, elevation, soil and land cover to produce a stratified map representing a set of 36 forest strata that have similar environmental characteristics and homogeneous forest structure (Additional file 1: SI.2, Fig. S2). Collecting a representative sample of lidar data for each stratum is crucial to estimate biomass at regional scales. While having a sample that is large enough to be statistically representative of an area [14], it is also crucial to rely on a methodology that provides unbiased estimates [15–18]. In this work, we used a stratified random sampling, recognized as one of the few unbiased methodologies to estimate biomass at a regional scale [15, 18]. The stratified map was used as a reference for the stratified random sampling with airborne lidar flights for inventory of forest structure. We designed lidar acquisitions using 49 flight lines with each flight line covering 500–2000 ha areas for a total of 83,000 ha of randomly sampled lidar data. The process of the lidar flight design followed the VT0005 methodology tool [18], taking into account the percent area of lidar coverage necessary, based on the number and area of strata to optimize the inventory sampling (Fig. 2). Flight lines were selected randomly in each stratum within the project nodes using the Reversed Randomized Quadrant-Recursive Raster (RRQRR) algorithm that is based on the implementation of the Generalized Random Tessellation Stratified (GRTS) algorithm [19]. The RRQRR toolbox allows for probability-based spatiality balanced sample designs to be implemented within a Geographic Information System (GIS). After the locations were selected, the flight lines were designed in three different sizes of 1-km width and with 5 km (500 ha), 10 km (1000 ha), or 20 km (2000 ha) length to allow the required coverage in each stratum and for cost-effective airborne data collection across several strata. The design allowed the airborne flights to be acquired in an optimum configuration with transects covering large areas and using less time for turns and realigning for flight headings. The orientation or heading of the flight lines were also selected randomly at 45° intervals. The lidar data were acquired using an Optech ALTM3033 at an elevation of about 1000 m, low enough to fly under clouds but high enough to cover relatively large swaths (500 m), with a point density of 4 returns/m^2^. Lidar scenes are for the most part located in lowland forests, with only 3 transects located in areas > 500 m asl elevation.

A canopy height model (CHM) was created for each lidar scene by taking the height difference between the digital surface model (DSM) and the digital terrain model (DTM) after normalizing the point clouds and posting the data at 1 m resolution (Additional file 1: SI.3). The CHM data were gridded at 100 m × 100 m cells by keeping the mean top canopy height (TCH) from 1 m^2^ pixels as the key lidar metric for developing biomass models and referred to as Lidar_TCH throughout the paper.

A total of 15 clusters of permanent tree plots were set in 15 of the lidar transects covering different strata in the BioREDD regions (Fig. 2). Each cluster plot has one permanent plot of 1 ha (100 m × 100 m) and eight ‘satellite’ plots of 0.25 ha (50 m × 50 m). The satellite plots are located at 250 m and 500 m away from the center permanent plots in four cardinal directions (Additional file 1: Fig. S3). This configuration aims to capture the structural and species composition variability of a larger area, and allowed [9] to develop the first local allometric equation for the region, which is used in this study. Each plot of a cluster is considered independent because spatial correlation length in tropical forests remains very small and was reported to become nonsignificant around 20 m [20]. The center location of the permanent plot was selected randomly in the lidar transect to allow sampling all forest conditions such as fragmented, degraded or secondary forests. In addition, 45 plots of 0.25 ha were systematically sampled in one of the lidar flight lines covering the terra firme and wetland forests. Field sites were mostly established in coastal areas at elevations < 100 m asl. Two sites were established above 200 m asl, in the Northern part of the study area (Chigorodo: 465 m, and Chontadural: 207 m). The implications of using field and lidar surveys distributed in three sub regions are addressed in the Discussion section. A total of 30,394 trees with diameter at breast height (DBH) ≥ 10 cm were measured using standard methodology for tropical forest inventory [21], with trees being tagged and identified botanically to species or morphospecies in the field for wood density quantification. Although a 10 cm DBH threshold is standard to estimate biomass in tropical forests, we acknowledge that not including smaller trees might have an impact on our biomass estimation of degraded areas, where a large number of small trees might be present. However, there is, to our knowledge, no data available to quantify the contribution of these small trees to the total AGB. Lianas were not sampled and the DBH of trees with buttresses was measured 50 cm above the buttress.

Wood density values were assigned to each tree using the Global Wood Density database for South America [22, 23], either at the species (12% of trees), genus (54%) or family level (24%). For all unidentified trees or of species absent from the database (10%), we assigned the mean wood density of the plot. The AGB was then estimated for each stem using two regional allometric equations, and the AGB values were summed over the plots [7] (see Additional file 1: SI.3 and Table S1, Eqs. S1, S2). Wood density information was used to determine the average wood density of the three forest classes used in our study, based on the forest class assigned to the plots they belong to: terra firme (25,861 trees, WD = 0.60 g cm^−3^), wetland (3918 trees, WD = 0.49 g cm^−3^) and mangrove (615 trees, WD = 0.79 g cm^−3^). The mean wood density of each class was assigned to the corresponding pixels of the LULC map. This wood density map product is referred to as “WD_map” (Fig. 2).

### Development of the lidar biomass estimator

We used geolocation of ground plots and extracted all 1 m lidar CHM pixel values that fell in the plots. These canopy height values were used to compute the lidar-derived mean top canopy height (TCH) for each plot. In addition to the 1 ha plots, the closest 0.25 ha plots were aggregated four by four in order to obtain ‘aggregated’ 1 ha plots. This resulted in a calibration dataset of 1 ha plots. We excluded cluster plots where 0.25 ha subplots were missing or were falling outside of a lidar scene, resulting in a cover area of less than 1 ha. However, these plots were used to estimate the mean wood density of each class.

We then developed a lidar-derived AGB model using the nonlinear least squares “nls” function in R [24], based on a total of 43 1 ha plots (15 real 1 ha plots, and 28 aggregated ones, Additional file 1: Table S1). Mean wood density of each plot (WD) and TCH were used in developing models [25, 26] that can be applied to all lidar data and mean wood density of each forest type:

1 $$AGB = a \left( {TCH \times WD} \right)^{b}$$

A leave-20%-out cross-validation with 1000 iterations was used to evaluate the model and determine its R^2^, RMSE and bias.

### Remote sensing predictors

Several remote sensing data were used as predictors in our random forest model. We used four Landsat8 bands: Red, Near Infrared (NIR) and two Short-wave Infrared (SWIR) bands covering the period of 2015–2016; two ALOS-2 PALSAR bands: HH and HV from 2015 acquisitions; and elevation from the Shuttle Radar Topography Mission (SRTM). Landsat provides information on forest type and canopy structure [27], ALOS PALSAR radar measurements at L-band wavelengths (~ 24 cm) capture the forest structure and biomass, separating low and high biomass areas [28], and SRTM imagery provides landscape elevation and measurements that are also correlated with forest height in degraded and fragmented areas [29] (see Additional file 1: SI.4 for more details). For pixels having no data in the ALOS layer due to topographical effects, the “surrogate” function available in RF algorithm allowed to predict H using the valid RS layers only, preventing holes or missing information in the final forest height map.

The images were co-registered and aggregated at 100-m spatial resolution. In addition to these 7 bands, we also created simple texture layers representing the local standard deviation of each band, based on a 5 by 5 pixel window from the original resolution of each dataset. Thus in total, 14 bands were used as an input for the RF algorithm and are referred to as RS predictors (see Additional file 1: SI.4).

### Mapping forest height and biomass

#### Random forest algorithm

The Random Forest (RF) machine-learning algorithm is an ensemble model of decision trees trained with selected number of features and a set of training data (lidar derived aboveground biomass) using a bagging technique [30]. RF algorithm has been used extensively to develop spatial estimates of the training quantity such as AGB across landscapes represented by different measurements from satellite imagery [31, 32]. Here, we use the RF algorithm in the form of regression trees based on the training data sets and remote sensing features that will provide estimates of forest height or AGB for image pixels in the form of the unweighted average of a collection of trees [18]. RF is often preferred to multiple regressions when a large number of predictors is used. It can handle multilinearity and detect outliers [33, 34]. The algorithm often provides reliable and unbiased estimates of forest height or AGB when the remote sensing data layers have strong sensitivity to a large range of biomass, and when the number of training data are large and widespread across the landscape within the study area [32]. However, at validation stage, the spatial accuracy of RF estimates must be quantified using an independent data set. Indeed, it is known that the RF algorithm tends to push the predictions towards the mean by overfitting when the data layers are noisy or have less sensitivity to the parameter of interest, thus failing to account for extreme values [32]. Here, we used the Matlab version of the random forest function “TreeBagger” and applied a bias correction approach developed to improve the dilution bias introduced by overfitting [32].

Lidar-derived canopy height and AGB were used as the response variable, while spaceborne remote sensing (RS) layers were the predictor variables in RF algorithm to predict forest height (H) and AGB over the study area. Our analysis indicated that creating a regional height map based on TCH from lidar (lidar_TCH layer) independently of the forest class and then converting this map to AGB using WD_map yielded more accurate results than predicting AGB directly from lidar-derived AGB (Additional file 1: SI.5, Table S2). We report the mean and standard deviation of H and AGB for each forest class: terra firme forest (including intact, degraded and secondary forest), wetland forest and mangrove.

#### Separating degrees of forest degradation

In addition to the three subclasses of terra firme forest mentioned above and based on the LULC map, we also report H and AGB across a forest degradation gradient, ranging from intact forest to severely degraded forest, based on a forest degradation index map (FDI) [35]. FDI is a remote sensing derived index, calculated for all pixels belonging to the terra firme class:

2 $$FDI = TCH + LCA + PC$$

where TCH is the top mean canopy height (m), LCA represents the percent area covered by large trees (H > 27 m, tree crown > 100 m^2^) [25] and PC is the percentage of vegetation cover (> 5 m). These three maps were developed using the same RF method as the height map, based on lidar data. The FDI product was trained over forests having different degrees of degradation to determine thresholds separating terra firme into four classes: intact, light, moderate to high, and severe degrees of degradation (see Additional file 1: SI.6). These classes were used as a post-classification tool to quantify H and AGB for different degrees of degradation of the terra firme forests.

### Uncertainty analysis

The uncertainty analysis includes a bootstrapping cross-validation approach using the RF model that provides a prediction map at the pixel level. We performed a leave-30%-out cross validation to assess the Height and AGB maps, where the lidar scenes are the sampling unit: at each iteration (N_it_ = 100), 30% (N = 14) of the lidar scenes are removed from the training sample to report the overall mean R^2^, RMSE and bias of all iterations. The iterations provided 100 height and hence AGB maps that were aggregated to provide the pixel level variance of the predictions.

However, the overall uncertainty associated with the AGB estimation of each pixel should also include uncertainty from lidar-biomass model and the ground-estimated biomass in addition to the RF prediction in a standard error propagation approach [36]:

3 $$\sigma_{total}^{{}} \left( u \right) = \sqrt {\sigma_{RS}^{2} \left( u \right) + \sigma_{modeling}^{2} \left( u \right) + \sigma_{field data}^{2} \left( u \right)}_{{}}^{{}}$$

where $\sigma_{total}^{{}} \left( {\text{u}} \right)$ is the uncertainty of the AGB estimate at the unit area (pixel), $\sigma_{RS}^{2}$ (*u*) is the error of the map for each pixel derived from the bootstrapping cross-validation, $\sigma_{modeling}^{2} \left( u \right)$ is the error associated with the lidar-derived AGB model, including the GPS error causing discrepancies between the lidar metrics and the field AGB, and $\sigma_{field data}^{2} \left( u \right)$ is the combined errors due to field measurements and biomass allometry. $\sigma_{field data}^{2} \left( u \right)$ was estimated based on a comparison of the DBH, H and WD measurements by two different teams in the field. The uncertainty related to measurements of DBH, H and wood density was used to estimate the error related to the allometric model, following [37] methodology, leading to an uncertainty of measurement and allometry of 21.4%. The calculation of the error for the height map was only based on the variance of the height map itself as the airborne lidar measurement errors can be considered relatively negligible.

The uncertainty of the AGB estimates for each vegetation class was estimated by integrating the pixel uncertainties described above over the regions of interest and accounting for the spatial correlation of the uncertainties as follows [36, 38]:

4 $$\sigma_{{}}^{2} \left( {class} \right) = \mathop \sum \limits_{i = 1}^{m} \mathop \sum \limits_{j = 1}^{m} cov\left( {\sigma_{ui,} \sigma_{uj} } \right) = \frac{1}{{m\left( {m - 1} \right)}}\left( {\mathop \sum \limits_{i = 1}^{m} \sigma_{ui}^{2} + 2\mathop \sum \limits_{i = 1}^{m} \mathop \sum \limits_{i < j}^{m} \rho \left( d \right)\sigma_{ui} \sigma_{uj} } \right)$$

where $\sigma^{2} \left( {class} \right)$ is the variance of the H or AGB estimates for the vegetation class of interest; *m* is the number of pixels; $\rho \left( d \right)$ is the spatial correlation function in terms of distance d based on a piecewise exponential semivariogram model; and $\sigma_{ui} \sigma_{uj}$ are the estimated variance associated with the height or AGB values at each pixel. We also used a ‘leave-one-scene-out’ cross validation scheme: at each iteration, we took one of the lidar scenes (49) out of the training data pool, and used it as testing data, similar to a jackknife cross-validation. This validation avoids spatial autocorrelation and assesses how the final map performs in areas where lidar data are unavailable. We evaluated our results in terms of the coefficient of correlation (R^2^) between the observed and predicted H or AGB, root mean square error (RMSE) and bias (mean difference between observed and predicted). For the leave-one-scene-out scheme, we report the results for the 49 iterations combined (total of testing points = number of lidar pixels), as well as the average R^2^, RMSE and bias of all iterations.

Finally, mean height and mean AGB of each vegetation class from the lidar “truth” were compared to the independently predicted data from all the leave-one-scene-out maps. We report the differences in mean height and AGB in each class in terms of percentage.

## Results

### Lidar-derived AGB model

**Fig. 3:**
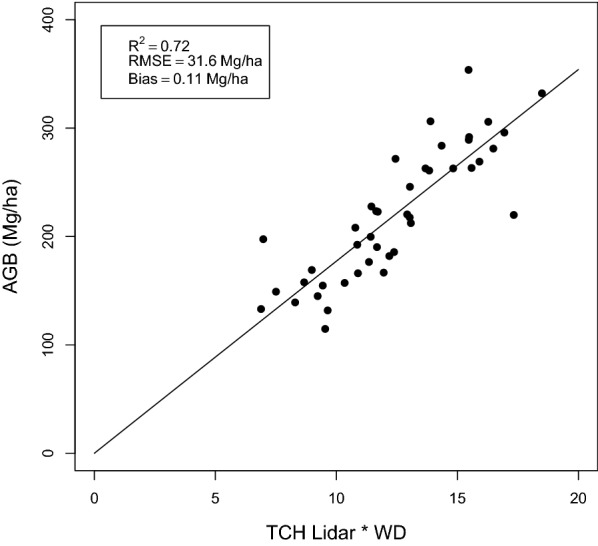
Lidar-derived AGB allometric model

The best model to infer AGB from lidar-derived TCH at 1 ha was:

5 $$AGB = 17.8 \left( {TCH \times WD} \right)^{1.0}$$

where WD is the plot-mean value of wood density (in g cm^−3^), and TCH is the plot-mean top canopy height (in m) from lidar observation (Fig. 3). We chose to use this power law model fit with exponent of approximately 1 (R^2^ = 0.72, RMSE = 31.6, Bias = 0.11) to stay consistent with previous studies, although a linear fit gave similar results (with a small intercept of 2.5 Mg/ha). In the following, we used Eq. (5) to estimate AGB, allowing to account for various forest types characterized by different mean wood density.

### Predictions of forest height and AGB

**Fig. 4:**
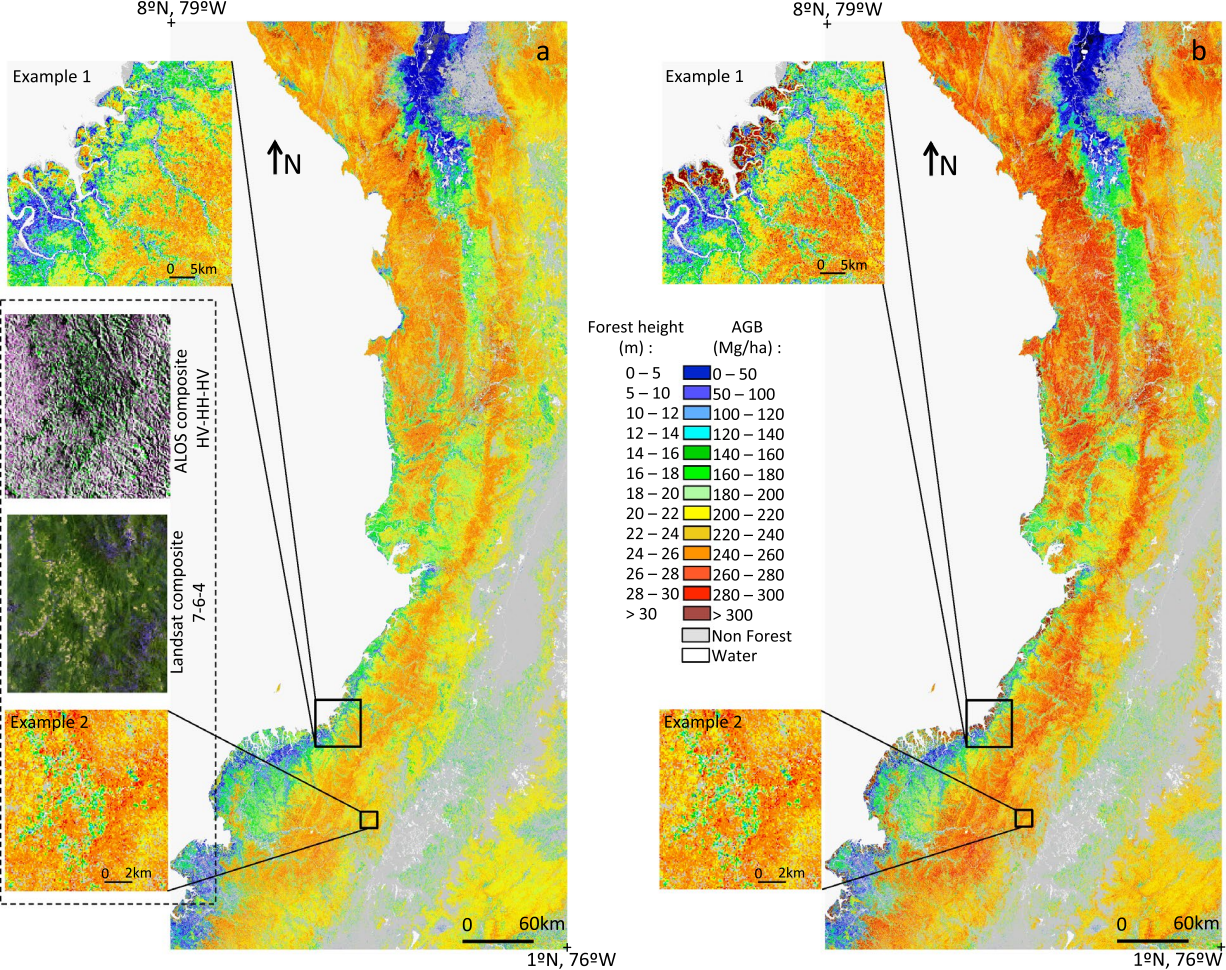
Height map (**a**) and AGB map (**b**). Example 1 shows how mangrove forest stands out in the AGB map because of its higher wood density. Example 2 displays the signature of fragmentation, as seen in ALOS and Landsat

Distribution of forest height and biomass predicted from the RF algorithms are shown in Fig. 4 at 100 m × 100 m (1 ha) gridded maps. The maps are color-scaled to show the variations of AGB and height estimates across the landscape separating the coastal vegetation from the interior terra firme and upland forests along elevational gradient, and intact old growth forests from degraded forests. The maps show a strong correspondence between height and AGB as expected from the lidar-biomass model. However, the patterns are also modulated by the wood density of forest types within each stratum, allowing for larger variability of AGB across the region.

Fragmentation and forest degradation are visible in both maps. These features are explained by the contribution of both ALOS and Landsat layers (Fig. 4, example 2). Results also show that intact forests are more homogeneous, with a standard deviation of 35 Mg/ha, compared to standard deviations around 60 Mg/ha for the two other classes.

Our maps present artifacts in some areas covered by lidar scenes (Fig. S5), due to the over-fitting of the RF model. RF tends to adapt closely to variations of the training sample, especially when a large number of trees are used to build the model [39]. We tested which parameters were optimal to get the best results in terms of R^2^, RMSE and bias and found that a number of 500 decision trees, one variable for random feature selection and a minimum of 13 observations per tree leaf were giving the best results. Reducing the number of trees might smooth out the lidar scenes from the maps but would also decrease the general accuracy. We chose to keep a high number of trees even though it makes these artifacts more visible.

### Regional statistics of AGB estimates

Across the region, mean height was 21.1 m: 21.8 ± 4.6 m in the terra firme class, 13.5 ± 7.9 m in the wetland class and 16.5 ± 6.4 m in the mangrove class (Fig. 4, Table 1), with low standard error in all classes (< 0.16 m). Mean AGB of terra firme forests was found to be 232.99 ± 49.4 Mg/ha, with 117.46 ± 68.5 Mg/ha in the wetland class and 229.91 ± 89.7 Mg/ha in the mangrove class (Table 1). Overall mean AGB was 224.31 ± 60.19 Mg/ha. AGB of the mangrove class is similar to AGB of the terra firme class: the lower mean height in the mangrove class was compensated by a higher WD (Fig. 4, example 1). Table 1 shows how the degradation gradient is reflected in its height and AGB distribution, ranging from 23.86 ± 4.12 m (or 254.79 ± 24.83 Mg/ha) in intact forest to 9.01 ± 4.62 m (or 96.24 ± 19.32 Mg/ha) in severely degraded areas. Within the terra firme class, degraded and secondary forests had lower mean height and AGB than intact forest (Table 1).

**Table 1:** Mean H and AGB and SE for all and vegetation classes

Metric	Vegetation class	Mean ± stdev	Standard Error (SE)	Area (ha)
*Classes derived from the LULC map*				
Height (m)	All	21.11 ± 5.47	0.06	10,681,445
	Terra firme	21.81 ± 4.63	0.06	9,702,404
	*Intact forest*	22.88 ± 3.26	0.06	6,116,808
	*Degraded *+* Secondary forest*	19.90 ± 5.84	0.07	3,585,596
	Wetland	13.52 ± 7.88	0.1	795,910
	Mangrove	16.45 ± 6.42	0.16	183,131
AGB (Mg/ha)	All	224.31 ± 60.19	2.58	10,681,445
	Terra firme	232.99 ± 49.40	2.77	9,702,404
	*Intact forest*	244.31 ± 34.82	2.94	6,116,808
	*Degraded *+* Secondary forest*	212.57 ± 62.40	6.86	3,585,596
	Wetland	117.46 ± 68.47	4.12	795,910
	Mangrove	229.91 ± 89.74	7.06	183,131
*Classes derived from FDI (terra firme only)*				
Height (m)	Intact	23.86 ± 4.12	0.06	7,171,530
	Degradation (all classes)	16.02 ± 4.49	0.06	2,530,874
	Light degradation	19.15 ± 2.67	0.06	1,147,469
	Moderate to high degradation	15.61 ± 3.19	0.06	923,431
	Severe degradation	9.01 ± 4.62	0.07	459,974
AGB (Mg/ha)	Intact	254.79 ± 24.83	2.98	*7,171,530*
	Degradation (all classes)	171.11 ± 49.32	2.25	2,530,874
	Light degradation	204.59 ± 15.70	2.63	1,147,469
	Moderate to high degradation	166.81 ± 30.54	2.35	923,431
	Severe degradation	96.24 ± 19.32	2.66	459,974

Fields in italics indicate which cover map (LULC or FDI) was used to determine classes

### Characteristics of forest degradation

The forest degradation classification based on FDI revealed that the area covered by intact forest is larger than what the LULC map indicates (~ 7 M ha instead of ~ 6 M). Their height and AGB were higher (but not significantly different), while degraded forest had lower height and AGB (but not significantly different). This is because some intact forest pixels were classified into degraded forest in the FDI map, and vice versa (Additional file 1: Table S3, Fig. S4). 84.1% of pixels classified as intact forest in the LULC map remained in the intact forest class in the FDI map, while 56.6% of the degraded or secondary forest pixels of the LULC map belong to the intact forest class in the FDI map (Additional file 1: Table S3). The overall confusion in the LULC map between intact and degraded forests suggest that the use of physical metrics such as the ones used in the FDI may be a more reliable approach to separate degraded from intact forests.

The new classification of degradation forest from the FDI approach provides large variations of forest biomass among degraded forests (Table 1). On average, forest degradation in Chocó region can reduce the mean biomass of intact forests by more than 30% but this varied from 19% for lightly degraded forests as in selective logging to about 62% for severely degraded forests. Our results provide, for the first time in this region, the ability to calculate the emission factors (changes of forest biomass from intact to any land use class) for different types and area of degradation for the region depending on the intensity of degradation.

### Uncertainty analysis

#### Uncertainty map and cross-validation

**Fig. 5:**
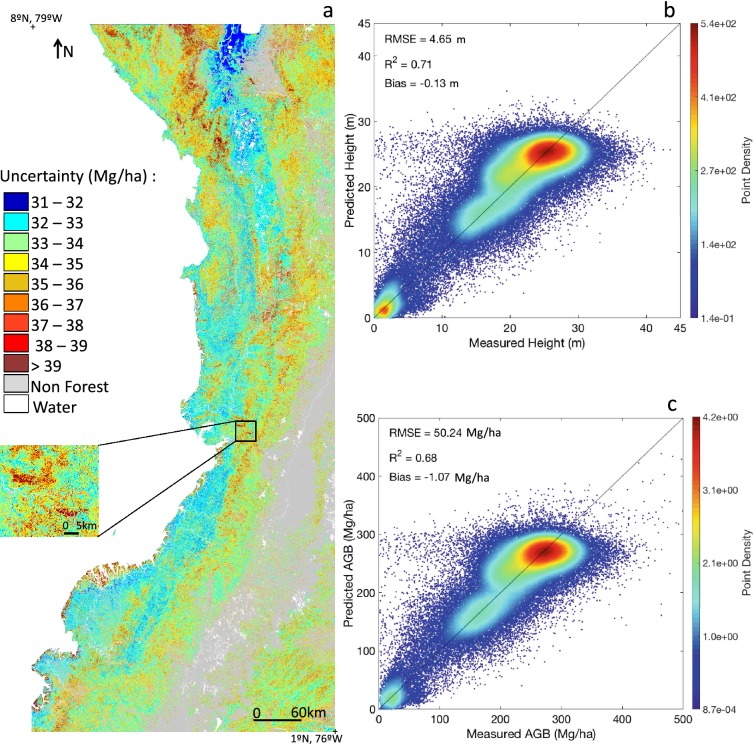
Uncertainty of AGB map, taking the error of the random forest mapping and the error of the lidar model. Example 1 shows how some lidar scenes show in the uncertainty map, because their prediction varies a lot depending on whether or not they were part of the training data for different iterations (**a**). Scatter plots of test samples based on the leave-one-scene-out cross validation: height (**b**), AGB form H (**c**)

The uncertainty map reported in Fig. 5a reflects the stability of our model. Uncertainty is higher when biomass is high, with more uncertainties in mangroves than in wetland forests (Fig. 5a). The pixels covered by lidar scenes have a higher standard deviation than their neighbors (Fig. 5a, subset). This is because taking a scene out results in predicted pixels that differ from the lidar predictions. The uncertainty map associated to the height map shows similar patterns and is not reported here.

Leave-30%-out cross-validation showed that height could be predicted with a R^2^ of 0.72, RMSE of 4.66 m and a bias of 0.11 m (Table 2). For AGB, the R^2^ was 0.68, RMSE was 50.66 Mg/ha and bias was 1.18 Mg/ha. Results for the leave-one-scene-out validation are also reported in (Table 2, Fig. 5b, c).

**Table 2:** R^2^, RMSE and bias of height and biomass with the one-scene-out cross validation (CV) and the leave-30%-out cross validation

Metric	CV method	R^2^	RMSE	Bias	R^2^ total	RMSE total	Bias total
H (in m)	One-scene-out	0.45	4.33	0.02	0.71	4.65	− 0.13
	30% out	0.72	4.66	0.11	–	–	–
AGB (in Mg/ha)	One-scene-out	0.45	46.45	0.74	0.68	50.24	− 1.07
	30% out	0.68	50.56	1.18	–	–	–

Leave-one-scene-out cross-validation revealed a large range of goodness of fit values in the height variable (R^2^ between 0.01 and 0.93; Additional file 1: SI.7, Table S4). Figure 5 and Additional file 1: Table S4 show a saturation of the predictive value of the model above ~ 25–30 m, leading to an underestimation of height for these pixels, and consequently of AGB. Pixels with a height of 25 m or higher represent 22% of the forested area, while pixels higher than 30 m represent less than 1%. Also, the two scenes having a mean elevation above 500 m were poorly predicted (Additional file 1: Table S4).

#### Vegetation classes uncertainties

Uncertainties related to each vegetation class are reported in Table 1 and were estimated using Eq. (2) based on semi-variograms (Additional file 1: Fig. S6).

The leave-one-scene-out validation also provided some insight on the uncertainty of each class within the lidar scenes: the regional map was found to predict average height and AGB well, with a difference of only 0.21% (Table 3). The model also had a good performance across most forest classes. Note that in wetland and mangrove forests the map under-predicted the variables by about 4%.

**Table 3:** Comparison of mean H and AGB in validation area

Metric	Class	Mean lidar “truth” ± stdev	Mean independent prediction ± stdev	Area (ha)	Difference in mean (%)
Height (m)	All	19.38 ± 7.97	19.34 ± 7.11	59,938	0.21
	Terra firme	20.84 ± 7.06	20.95 ± 5.88	48,824	0.51
	*Intact forest*	23.03 ± 5.16	22.89 ± 4.14	28,727	0.21
	*Degraded *+* Secondary forest*	17.83 ± 7.23	18.26 ± 6.64	21,336	2.41
	Wetland	12.16 ± 8.59	11.64 ± 7.81	9563	4.30
	Mangrove	18.14 ± 6.41	17.39 ± 5.12	1551	4.11
AGB (Mg/ha)	All	204.67 ± 87.28	204.25 ± 78.47	59,938	0.21
	Terra firme	222.54 ± 75.44	223.72 ± 62.77	48,824	0.53
	*Intact forest*	245.98 ± 55.13	244.5 ± 44.16	28,727	0.60
	*Degraded *+* Secondary forest*	190.40 ± 79.02	195.05 ± 72.98	20,204	2.44
	Wetland	105.66 ± 74.62	101.08 ± 67.82	9563	4.33
	Mangrove	253.52 ± 89.63	243.06 ± 71.60	1551	4.13

## Discussion

### Critical steps in mapping AGB

Our results suggest that developing a map of forest height directly from the lidar data before converting the heights to AGB improves the estimation accuracy (Additional file 1: SI.5). Lidar-biomass models may introduce uncertainty in the biomass estimation that may be propagated spatially into the map through the machine learning approach. The height map is produced from extrapolating the observation of spatial remote sensing data from airborne data to the entire region using satellite based remote sensing data that are sensitive to height, canopy structure and their spatial variations. Furthermore, by producing a map of forest mean canopy height or structure, it is easier to implement different estimators of AGB and further improve the AGB map if more ground data are collected or new models are developed. Both the lidar-derived AGB model and the wood density map can be updated in the future and be applied on the regional height map.

The biomass estimation from AGB models depends on the mean wood density values at the plot scale. We used the global wood density database (GWDD) [23] to obtain wood density of each vegetation class based on the identified trees of the field data. We compared our results to a similar method based on average wood density of each site from a recent study that uses a branch to stem wood density relationship from a selected number of species [9]. This method led to a similar lidar-derived AGB model, with a mean difference in estimated AGB of 0.66 Mg/ha and maximum difference of 3.69 Mg/ha in all field plots. However, when using a different mean wood density for each class of vegetation for converting the height map to biomass, the results may be different. For example, the sampling approach gives a WD of 0.562 for the terra firme class, 0.65 for mangroves, and 0.538 for wetlands, instead of 0.60, 0.785, and 0.485, derived from global datasets respectively, leading to a − 5.5% difference in AGB across the whole map (− 8.7% in terra firme forest, − 17.0% in mangrove forest and + 10.7% in wetland forest).

Furthermore, we used the same plot level mean wood density for intact, degraded and secondary forests, although degraded and secondary forests often have lower wood density values in this region [40]. By overestimating the biomass of degraded forests due to higher wood density, the emissions from forest loss and degradation may be underestimated. The variation of wood density across elevation gradients and successional range is another source of uncertainty. For instance, [25] showed that large trees in Chóco had a higher wood density than small trees. It has also been shown that elevational variations of wood density in the equatorial Andes were slight but significant [41]. For these reasons, it would be good to address this point in the Chocó region in the future.

While our ground dataset is relatively large in number of plots and plot size for this type of study relating field plots to lidar metrics, it only covers the dominant forest types, therefore potentially misrepresenting the diversity of environmental conditions in the area. Moreover, we had more plots, and therefore more trees, located in terra firme forest than in wetland forests and mangroves, increasing the uncertainty for these two classes. Finally, the land cover map we used to assign the right wood density to each pixel might also have some errors that can propagate to the estimation of AGB. For instance, wetland forest and mangroves being often adjacent, there is a risk of misclassification that is difficult to avoid and can give different results using Eq. 3, with WD_wetland_ = 0.49 g/cm^3^ and WD_mangrove_ = 0.79 g/cm^3^. Since approximately 90% of the forested area belongs to the terra firme class, the overall uncertainty of the map is not expected to be significantly affected by the uncertainty of these two classes.

Mangroves were found to have a high mean AGB even though their mean height is lower than the one of the forest class, emphasizing the importance of including wood density as a parameter of the AGB model, in addition to height. Wetland forests have the lowest mean AGB, since they are characterized by a lower TCH and a lower wood density, due in part to the presence of palm species [9]. These forests are dominated by the Myristicaceae family, largely traded in the region to produce commercial wood.

### Carbon storage potential of degraded forests

We described an approach to create and validate a height map and an AGB map at 100 m resolution in the Chocó region of Colombia. Such maps are essential to the BioREDD project and represent one of the first steps towards assessing the natural resources of the Chocó region. The biomass map is a tool providing important baseline information on the state of the Chocó forests, highlighting issues regarding deforestation and degradation. Degraded forests represent 37% of the terra firme class, or over 3 million hectares, and had on average an AGB 23% lower than intact terra firme forests (33% when relying on the FDI classification). This converts to an estimated biomass loss of 115 million Mg across the region (or 212 million Mg when relying on FDI). Although the study area is one of the least developed in Colombia, the fact that 37% of the pixels of terra firme forests are classified as degraded suggests that humans already have a massive impact on this ecologically important region. Forest removal, associated to the high amount of rainfall in the area (> 4000 mm years^−1^), leads to impoverished soils and low-productivity agricultural and cattle-ranching systems. Knowing where these areas are located and how severe the damages are helps address degradation issues and focus efforts on these areas to avoid further deforestation. Efforts to develop alternative non-timber forest exploitation models will be paramount to protect forest functioning and biodiversity. This map could also be an important tool in future biodiversity mapping of the region, considered a biodiversity hotspot.

In addition to the LULC map, we proposed an alternative method relying on a forest degradation index based on canopy height, forest percent cover and presence of large trees. Similar FDI indices could be used in other forests and become a standard way to classify forest degradation, since they are based on the general assumption that an intact forest should have a relatively high canopy, a high percent cover and large trees. On the opposite, a degraded forest is more likely to have a lower canopy height, a low forest percent cover and less large trees.

Our maps can be used as a benchmark for people involved in programs such as BioREDD and other stakeholders, to better grasp the potential of the region in term of its forest resources and guide them in their decision-making. The main goal of the BioREDD project was to help local communities drive sustainable development, in part based on carbon revenues and sustainable agricultural practices. This biomass map, along with basic GIS tools, will help them quantify the amount of carbon specific areas hold, and locate areas that would be the most favorable to the development of agriculture with a minimal impact on the forest and its biodiversity. Such biomass maps, developed following the required IPCC guidelines, are the first step toward potential future projects such as REDD, which require regions or countries to report on the present state of their forests. Although these programs strongly depend on developed countries to be implemented and be successful, providing high quality biomass products is essential for regions such as Chocó.

This work shows how airborne lidar data can be used to complement limited ground data, which is very helpful in remote areas where ground data are difficult to collect on a large scale, such as in the Chocó region [26]. The large number of lidar pixels used as the calibration dataset allowed us to compensate for the relatively limited inventory data that was collected. The high coefficient of correlation between inventory-estimated AGB and lidar-derived TCH and low bias allowed us to develop a reliable model able to convert forest height to biomass over the whole Chocó region.

### Distribution of ground and lidar measurements

The BioREDD project at the origin of this study focused on three sub-regions of the Chocó Biogeographic corridor, where lidar data and field data have been collected [12]. This clustering of data in three areas may have had an impact on the results, especially if the forest structure of the areas that were not covered by lidar is different from the one of these three regions. Our uncertainty analysis, especially through the leave-one-scene out validation, allowed us to have a more realistic estimate of uncertainty over areas where there was no ground or lidar data. Although these tests were made within the three regions of interest of the Bioredd project, this was the best way we found to estimate the overall uncertainties in the region. Without any other data sets in the in-between regions, we cannot evaluate the uncertainty better than our simulations from the machine learning and existing lidar samples. Future research in the Chocó region should focus on adding field data and/or lidar scenes in these in-between areas in order to have a more homogeneous coverage of the region and to validate our results in large areas where no lidar data have been acquired.

### Improvements on uncertainty estimates

We introduced a new cross-validation method based on removing sequentially a single lidar scene. This approach follows closely the jackknife cross-validation concept [42], and is an effective way to test how the model is performing in areas not covered by lidar data. This method provides more insights on the lidar sampling approach and how to improve the sample size and area covered by lidar for future monitoring of forest structure and biomass. It also provides a more conservative evaluation of the performance of the map, with a lower mean R^2^ across iterations (Table 2).

The leave-one-scene-out cross-validation also allowed us to highlight specific issues: the goodness of fit varied greatly across scenes depending on the variations of TCH and the landscape topography. We found that the uncertainty of the map was significantly larger in areas of TCH > 30 m where the sensitivity of the satellite data to extrapolate the lidar height over the landscape was less. The uncertainty of the map is also larger in higher elevation because most of the training data from airborne lidar are in low elevation and around the coastal forests where the REDD projects were concentrated, leading to larger ambiguity in representing forests across steep slopes (Additional file 1: Fig. S7). Users should therefore keep in mind that the AGB map has a large uncertainty in high elevation areas.

Future remote sensing data from BIOMASS and NASA-ISRO Synthetic Aperture Radar (NISAR) missions with improved relation to forest structure can help reducing the uncertainty in high biomass and dynamic forests. BIOMASS is a P-band radar mission led by the European Space Agency (ESA) that will launch in 2023 and will map forest height and biomass at a resolution of 200 m. The BIOMASS instrument operates at a wavelength long enough to penetrate the structure of dense tropical forests such as Chocó. NISAR is a L-band radar mission that is set to launch in early 2022 and will, among other things, estimate forest biomass globally at a spatial resolution of 100 m. NISAR will be most useful to track the temporal changes of forest degradation, with a repeat pass of 6 to 12 days.

## Conclusion

The maps produced in this study provide the variations of forest structure and the aboveground biomass, particularly in lowlands of the Pacific coast of Colombia and for the first time, captures the impacts of forest degradation in the poorly known Chocó region. The maps can be used as a new reference for reporting on the state of the forest for regional emission reduction activities and REDD+ projects, making it a powerful tool for decision-making. The uncertainty of the maps representing the lowland forests was relatively low, suggesting the methodology of systematic lidar inventory of forests a powerful tool to be used for the other regions of Colombia or South American rainforest. The lack of systematic ground forest inventory plots in the region has prevented large scale research on the forest structure, biomass and the rich ecological diversity of forests of Chocó or the Colombian Amazon. Our approach has already been implemented in other countries for national forest inventory in the Democratic Republic of Congo (DRC) [26], Kalimantan [35] and Brazil [43].

Our results also suggest that despite the low uncertainty of the derived forest biomass in lowland land cover classes, the uncertainty over areas without lidar and ground samples, or high elevation remains relatively large. Improving lidar and ground sampling in these regions and using advanced remote sensing observations such as terrain corrected radar imagery may improve the mapping approach in these regions.

## Additional file

**Additional file 1.** Detailed information on LULC map, stratification map, field data, remote sensing predictors, FDI map and methodology.

## Abbreviations

AGB: aboveground biomass
ALOS–PALSAR: Advanced Land Observing Satellite–Phased Array type l-band Synthetic Aperture Radar
asl: above sea level
BioREDD: Biodiversity-Reduced Emissions from Deforestation and Forest Degradation
CHM: canopy height model
CV: cross validation
DBH: diameter at breast height
DRC: Democratic Republic of Congo
DSM: digital surface model
DTM: digital terrain model
ESA: European Space Agency
FDI: Forest Degradation Index
GIS: Geographic Information System
GRTS: Generalized Random Tessellation Stratified
GWDD: Global Wood Density Database
H: height
HH: horizontal–horizontal
HV: horizontal–vertical
LCA: large canopy area
Lidar: light detection and ranging
LULC: land use land cover
TCH: mean top canopy height
NIR: near infrared
NISAR: NASA-ISRO (Indian Space Research Organisation) Synthetic Aperture Radar
PC: percentage of vegetation cover
R^2^: coefficient of determination
REDD: Reducing Emissions from Deforestation and forest Degradation
RF: random forest
RRQRR: Reversed Randomized Quadrant-Recursive Raster
RMSE: root mean square error
RS: remote sensing
SWIR: short-wave infrared
SRTM: Shuttle Radar Topography Mission
stdev: standard deviation
WD: wood density
