# Supplementary material for: Forest degradation and biomass loss along the Chocó region of Colombia
Source: Carbon Balance Manag. 2019 Mar 23;14:2. doi: 10.1186/s13021-019-0117-9 (PMC6446973; doi:10.1186/s13021-019-0117-9)
Supplement: Supplementary file 1 — Additional file 1. Detailed information on LULC map, stratification map, field data, remote sensing predictors, FDI map and methodology. [file 13021_2019_117_MOESM1_ESM.docx]

**SI.1 Land Use and Land Cover Map (LULC)**

The LULC map for the BioREDD region has been developed using time seires of Landsat imagery from 1998-1991, 1999-2001 and 2010-2012 at 30 m resolution. Landsat imagery have been mixed with 2007-2010 ALOS PALSAR data at 25m resolution to help with cloudy pixels and classifying inundated vegetation and forest degradation/fragmentation through a decision tree approach. The Classification has been performed using a Maximum Likelihood Classification approach, using training and validation data based on a combination of lidar derived canopy cover and height. Degraded forest was defined with the threshold of height > 5 m (forest definition) and tree cover between (30-75%), based on observations in the field and lidar data analysis. Intact forests in this region was found to approximately have more than 75% tree cover. In addition to this definition, we allowed the time series classification of Landsat to determine if a pixel was spectrally changed from 1990 to 2000 and from 2000 to 2007 and 2007 to 2012 in our analysis. If the forest pixel was not changed regardless of the tree cover, we assumed the forest remained intact through time. The final map used in our study, therefore, only include degradation pixels that occurred between 1990 to 2012. An accuracy assessment was performed using 1000 random samples over intact, degraded and non-forest. The overall classification accuracy was reported to be 91%, with a mapping accuracy of 90% for intact forest, and 74% for degraded forest and non-forest.

The LULC map was validated by BioREDD (https://www.climatelinks.org/resources/bioredd-colombia) and includes 14 classes as shown below:

**
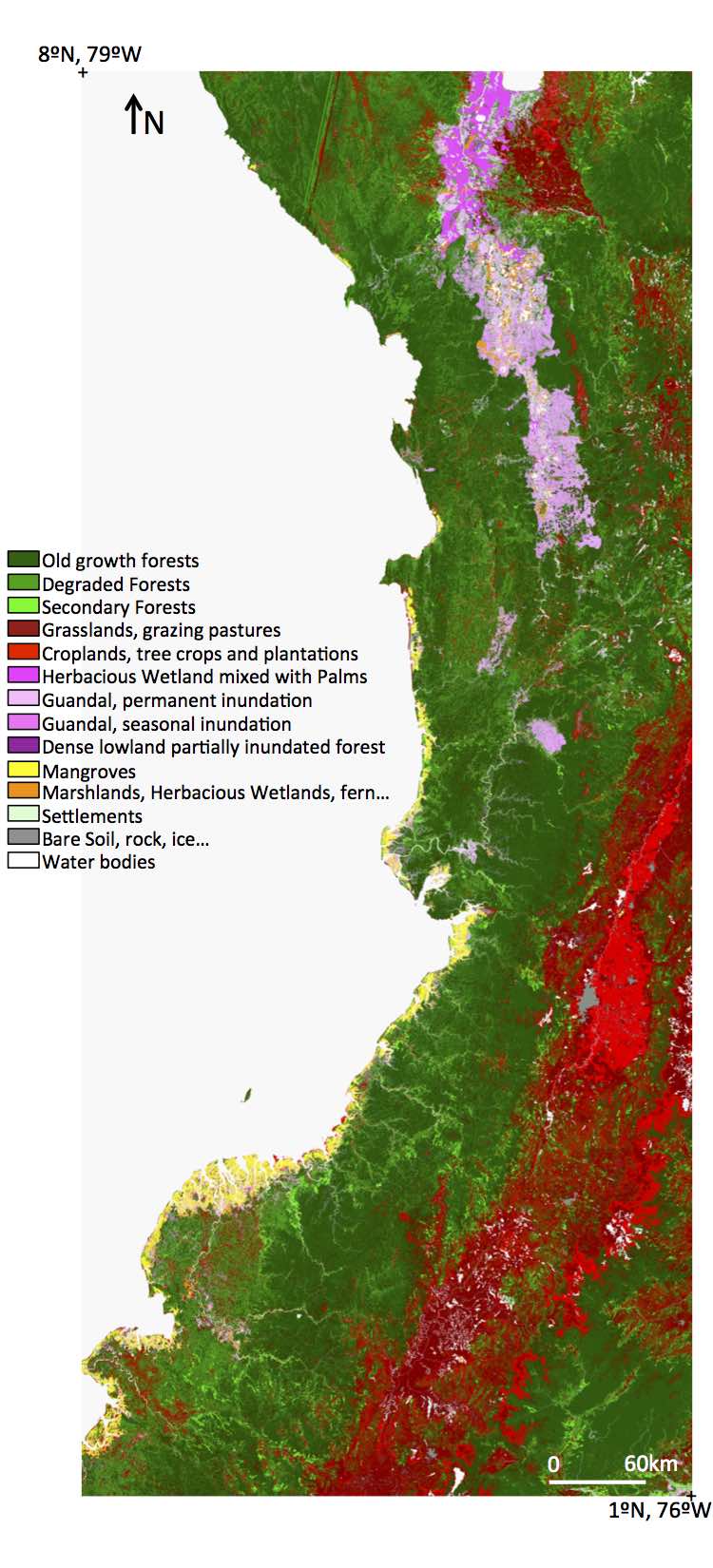
**

**Fig.S1: Land Use Land Cover map produced for BioREDD.**

**SI.2 : Stratification map**

The stratification map was created by combining four datasets:

**Climate data:** We used a 1km resolution map representing the rainfall of the driest quarter of the year from the bioclimatic variables developed by Hijmans et al. (2004, <http://biogeo.berkeley.edu/>). This metric is known to be the most important indicator of forest structure and dynamics in tropical regions (Malhi et al., 2008). Although annual rainfall in the Pacific region of Colombia can reach above 10,000mm, there is some variability along the latitudinal and elevational gradients. We used three categories to separate the rainfall of driest quarter by dividing them into areas < 300 mm (class 1), 300-600 mm (class 2), and >600 mm (class 3) rainfall.

**Elevation Data:** The topography layer was based on Shuttle Radar Topography Mission (SRTM) data. SRTM is a single-pass interferometry synthetic aperture system (SAR) that acquired data in 2000 and was resampled at 100m resolution. Three categories were used to distinguish coastal areas from hilly inland areas and higher elevation areas on the foothills of the Andes. The segmented classes are 1-100 m (class 1), 101-600 m (class 2), and >600 m (class 3) elevation.

**Soil Data:** The soil class data have been derived from the Soil and Terrain Database for Latin America and Caribbean (SOTERLAC, version 2) at 1:5 million scale (Dijkshoorn et al., 2005). The assignment of the soil class was based on matching the descriptions of the map units and comparing with the landforms and geographical description provided by Sombroek (2000). The categories were reduced to only 10 classes to focus on the most important categories for forest types and dynamics, of which 8 were found in the area of study. The classes included: heavily leached white sand soils (class 1), ancient oxisols (class 2), more recent oxisols (class 3), less infertile lowland soil (ultisols and entisols) (class 4), alluvial deposits from the Holocene (class 5), contemporary alluvial deposits (class 6, 8 and 9).

**Land Cover:** We created a land cover map based on ALOS PALSAR HH and HV acquired in 2007-2010, at 100m resolution, separating the landscape into 3 classes: Forest (class 1), Swamp (class 2) and Non-Forest (class 3). ALOS is using L band and is sensitive to forest type and structure and is not affected by cloud cover.

The climate and soil data were resampled to 100m resolution to match the other layers. The stratification was performed by multiplying all data layers to create 3 x 3 x 3 x 10=270 classes in IDL (Interface Description Language, Exelis). Only 103 of these strata are located in the coastal region, with only 50 classes covering more than 95% of the entire image area. The classes representing the last 5% of the study area were gathered in one single class. 36 classes fell into the three BioREDD regions of interest and were used to capture the biomass variations by lidar sampling (https://www.climatelinks.org/resources/bioredd-colombia).


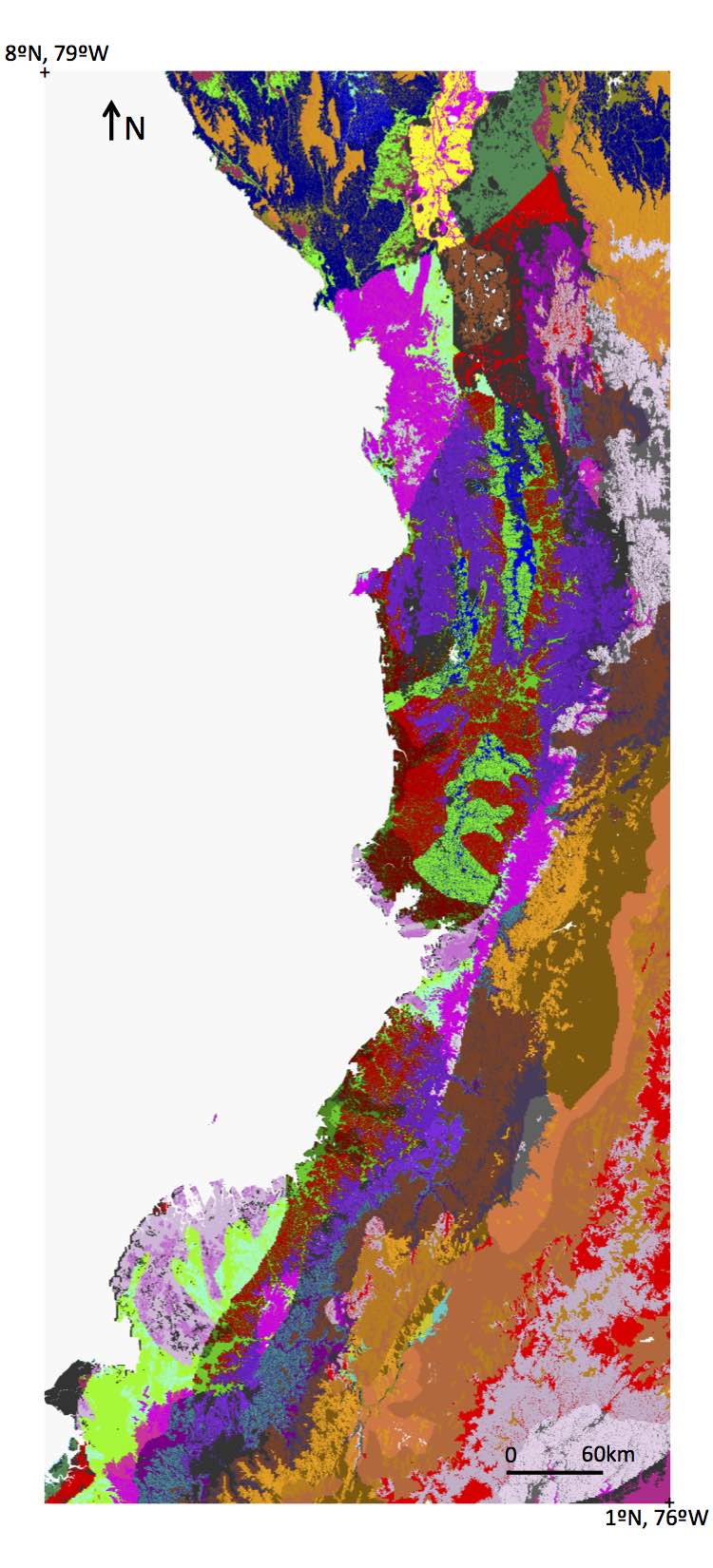


**Fig.S2**. Landscape stratification map (left). Each color represents a different stratum (right).

**SI.3 : Lidar data processing and estimation of AGB from field data and**

**Lidar data processing:**

Discrete return lidar data were collected between June and November 2013 using a LaserScan Optech 3033 laser on board a Cesna airplane. Flight altitude was 1000m, using a scan angle of ±20º and a pulse frequency of 30 kHz, resulting in a point density of 4 returns/m^2^. The lidar data was processed and rasterized to create a Digital Terrain Model (DTM) from points classified as ground and from points classified as vegetation to create a Digital Surface Model (DSM), at 1m resolution.

**AGB estimation from field data:**

Tree measurements in all plots included all trees with DBH ≥ 10 cm in diameter, with trees being tagged and identified by species for wood density assignment. A minimum number of 50 trees in different DBH classes were selected for height measurements. The number of trees with height measurement represented between 7 and 16% of the total number of trees registered in each site. All trees in San Pablo already had height measurements. A tree-diameter height (H-D) predictive model developed as part of the project for the same area was used to estimate the height of the trees that did not get measured in the field (Duque et al., 2017). Finally, AGB of each plot was estimated using two regional allometric equations based on Duque et al. (2017), one for trees (Eq S1) and one for palms (Eq S2).

$AGB={0.089\times({DBH}^{2}\times H\times WD)}^{0.951}$ (Eq S1)

$AGB={0.841 \times({DBH}^{2} \times H \times DMF)}^{0.7}$ (Eq S2)

Where DBH is diameter at breast height or above buttress, H is the total height of a tree, WD is the wood density in g/cm^3^ and DMF is the dry mass fraction, which is the equivalent of wood density for palms. Note that a total of three satellite plots have not been measured and that an additional 21 plots did not fall into the Lidar scenes or had some obvious geolocation errors, including 4 of the 45 plots. These plots were not considered in our studies, giving a final total of 15 plots of 1ha and 142 plots of 0.25ha.


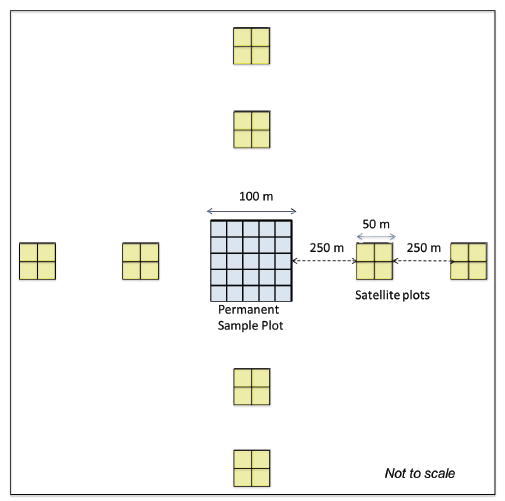


**Fig.S3**. plot design of a permanent plot and its 8 satellite plots

**Table S1**. List of the 43 plots used to calibrate the lidar AGB model. TCH from lidar and AGB estimated from ground data and wood density of each plot are given. M: Mangrove, T: Terra firme, W: Wetland

| site | permanent/sat | TCH lidar | AGB | WD |
| --- | --- | --- | --- | --- |
| Acapa (M) | permanent | 23.70 | 219.86 | 0.73 |
| Bajo Mira (W) | permanent | 19.41 | 114.73 | 0.49 |
| Buenavista (T) | permanent | 25.73 | 295.88 | 0.66 |
| Río Cajambre (W) | permanent | 16.05 | 197.51 | 0.44 |
| Bajo Calima (T) | permanent | 18.67 | 190.22 | 0.63 |
| Bajo Calima 2 (T) | permanent | 20.88 | 220.36 | 0.62 |
| Chigorodo (T) | permanent | 24.89 | 260.84 | 0.56 |
| Concosta (T) | permanent | 24.18 | 289.16 | 0.64 |
| Chontadural (T) | permanent | 27.43 | 269.05 | 0.58 |
| Pizarro (T) | permanent | 24.95 | 306.25 | 0.56 |
| Río Pepe (T) | permanent | 18.80 | 157.16 | 0.55 |
| Río Pepe 2 (T) | permanent | 19.90 | 208.11 | 0.54 |
| San Pablo (T) | permanent | 24.95 | 263.22 | 0.62 |
| Río Cajambre (W) | sats_1234 | 16.73 | 169.09 | 0.54 |
| Río Cajambre (W) | sats_5678 | 20.89 | 222.86 | 0.56 |
| Curvarado (T) | sats_1234 | 24.40 | 353.68 | 0.63 |
| San Pablo (T) | sats_1234 | 22.26 | 283.76 | 0.64 |
| San Pablo (TF) | sats_5678 | 20.42 | 212.34 | 0.64 |
| Concosta (T) | sats_1234 | 23.13 | 262.78 | 0.64 |
| Bajo Calima (T) | sats_1234 | 19.30 | 227.68 | 0.59 |
| Bajo Calima (T) | sats_5678 | 16.90 | 131.81 | 0.57 |
| Río Pepe (T) | sats_1234 | 22.96 | 245.84 | 0.57 |
| Río Pepe (T) | sats_5678 | 20.66 | 199.71 | 0.55 |
| Chigorodo (T) | sats_5678 | 22.37 | 271.65 | 0.56 |
| Bajo Mira (W) | sats_1234 | 14.30 | 133.08 | 0.48 |
| Bajo Mira (W) | sats_5678 | 14.69 | 149.09 | 0.51 |
| Bajo Calima 2 (T) | sats_1234 | 21.25 | 217.59 | 0.61 |
| Bajo Calima 2 (T) | sats_5678 | 19.77 | 185.64 | 0.63 |
| Río Pepe 2 (T) | sats_1234 | 26.59 | 291.77 | 0.58 |
| Río Pepe 2 (T) | sats_5678 | 21.06 | 223.35 | 0.55 |
| Buenavista (T) | sats_1234 | 24.70 | 305.80 | 0.66 |
| Buenavista (T) | sats_5678 | 28.20 | 332.11 | 0.66 |
| Chontadural (T) | sats_1234 | 28.38 | 281.08 | 0.58 |
| 45parcelas (T) | 1 | 21.03 | 181.96 | 0.58 |
| 45parcelas (T) | 2 | 20.40 | 166.65 | 0.59 |
| 45parcelas (T) | 3 | 19.05 | 176.49 | 0.60 |
| 45parcelas (T) | 4 | 14.35 | 139.17 | 0.58 |
| 45parcelas (T) | 5 | 15.25 | 144.99 | 0.61 |
| 45parcelas (T) | 6 | 15.64 | 154.75 | 0.60 |
| 45parcelas (T) | 7 | 15.00 | 157.71 | 0.58 |
| 45parcelas (T) | 8 | 18.91 | 166.09 | 0.58 |
| 45parcelas (T) | 9 | 18.71 | 192.38 | 0.58 |
| 45parcelas (T) | 10 | 22.45 | 262.80 | 0.61 |

**SI.4 Remote sensing predictors**

Remote sensing data from three different spaceborne sensors were mosaicked for the same 7º by 3º area and used as predictors in our random forest model: 4 Landsat8 bands (Red, Near Infrared (NIR) and 2 Short-wave Infrared (SWIR) bands), 2 ALOS PALSAR bands (HH and HV) (https://www.asf.alaska.edu/sar-data/palsar/) and elevation from SRTM (https://www2.jpl.nasa.gov/srtm/).

We created a Landsat8 mosaic using Google Engine and an embedded algorithm that masks clouds and replace them by valid pixels from other images (https://earthengine.google.com). For each pixel, we used the mean of all the landsat8 images available between 2013 and 2016. The mosaic has a resolution of 0.00025º, which corresponds to approximately 30m close to the equator, and includes the last four bands of Landsat (4 to 7) : Red, Near Infrared (NIR) and 2 Short-wave Infrared (SWIR) bands (Landsat bands 5 and 7).

We used the SRTM v3 data released by the National Aeronautics and Space Administration (NASA) in 2014 at a resolution of 1 arc second, or 0.000278º or approximately 30m near the equator. The SRTM v3 data have been void-filled with elevation data from ASTER GDEM (Global Digital Elevation Model Version 2).

The Phased Array type L-band Synthetic Aperture Radar (PALSAR) instrument operates at 1270 MHz, allowing the sensor to see through clouds and to not be affected by the atmosphere. The products used in this study are from data collected between 2007 and 2010 and have a resolution of 0.000222º. They are slope-corrected and orthorectified using SRTM v3 data. They are also calibrated to avoid striping effect between neighboring strips and equalize their intensity differences (Shimada et al., 2009). More recent data from 2015 were excluded from our dataset because of missing strips in the study area. The ALOS products also come with a data mask that excludes water bodies and areas with high slopes that present some issues caused by shadows and are badly interpolated.

In the event of a missing/masked pixel value in one or several predictor layers, RF automatically relied on the other layers to predict a pixel, using the “surrogate” option of the RF function in matlab. In this study, only ALOS had some missing data due to shadows in rough topography areas. SRTM missing pixels where replaced by ASTER data.

Our preliminary results testing which input layers should be used as predictors showed that using all the available data layers was giving better results, in accordance to results of a recent study (Xu et al., 2016). We also followed the methodology of this study to address the bias introduced by the averaging process of RF, leading to the overestimation of small values and underestimation of high values. This bias correction is a modified version of a bootstrap correction method (Hooker & Mentch, 2015) involving two runs of RF to get a bias-corrected prediction. For more details, see Xu et al. (2016).

**SI.5 AGB map from height map vs AGB map from lidar AGB**

We tested two approaches to estimate AGB at a regional scale. The first approach consisted in first creating a wall-to-wall height map using RF at the regional scale, based on lidar-derived TCH at 100m resolution (Lidar_TCH) and the remote sensing layers. Once the regional height map was produced, the lidar-derived AGB model was applied to the whole region using Eq 3. The result is an AGB map of the whole study area. This approach has the advantage to not rely on local allometry and knowledge of wood density prior to mapping the region. Besides, producing a height map can have other useful purposes, such as studying the structure of the forest itself and looking for disturbances caused by fires or blow downs, for instance. The second approach consisted in using Lidar_AGB to predict AGB directly at a regional scale using RF, without creating a height map first. This approach has been used in several regional mapping of biomass using machine-learning algorithms (eg. : Rodríguez-Veiga et al., 2016; Saatchi et al., 2011).

Predicting AGB directly from the AGB lidar layer gave a slightly lower R^2^ of 0.66, a similar RMSE of 50.49 Mg/ha and a higher bias of -2.54 Mg/ha than predicting AGB from the height map obtained with RF, compared to the results given by the first approach in the paper. Both the average R^2^ from all iterations and the R^2^ based on the validation from all the plots together in the same graph were higher when predicting AGB from height instead of predicting it directly from AGB (Table S2). These results indicated that estimating AGB from the height map was the best method and we focused the rest of our analysis on that product. Results infering AGB from the height map are presented in the main paper (Fig3).

**Table S2.** R^2^, RMSE and bias of biomass from lidar AGB with the one-scene-out cross validation and the leave-30%-out cross validation

| Metric | CV method | R^2^ | RMSE | bias | R^2^ total | RMSE total | bias total |
| --- | --- | --- | --- | --- | --- | --- | --- |
| AGB from Lidar AGB (in Mg/ha) | One-scene-out | 0.40 | 48.15 | -0.13 | 0.66 | 51.56 | -1.45 |
|  | 30% out | 0.64 | 50.49 | -2.54 | - | - | - |

**SI.6 Mapping of Forest Degradation Index (FDI)**

We developed a wall-to-wall map of forest degradation (1 ha resolution) to better quantify the extent and degree of degradation of the forests of the region. We define FDI as a function of mean top canopy height (TCH), large trees canopy area (LCA) and forest percent cover (PC) as defined in Eq.2., such that FDI=TCH+LCA+PC. The LCA and PC maps were produced the same way the height map was produced, using RF to produce wall-to-wall maps of the two metrics. All the Lidar scenes, converted to PC and LCA, were used as training data, and the RS layers were used as predictors. PC represents the percentage of pixels >5 m within a 1ha pixel, based on the 1m Lidar canopy height model and agreeing with the guidelines of the CDM of the Kyoto Protocol (UNFCCC, 2002), defining a forest as an area covered by vegetation higher than 5 meters and with PC larger than 30%. LCA is the percentage of area covered by pixels >27 m and gathered in clusters of at least 100m^2^ (here 100 pixels of 1m in the Lidar canopy height model) (Meyer et al., 2018). Here, the TCH map was normalized between 0 and 100 to calculate FDI.

The FDI map (Fig.S4) was further segmented into 5 classes after a cross examination of the FDI map, the 1m lidar CHM and the original satellite images. We defined the following classes: intact forest (FDI>160), light forest degradation (140<FDI<=160), moderate to high forest degradation (140<FDI<=100) and severe forest degradation (FDI<=100).

**Fig.S4**: Forest degradation classes based on the forest degradation index (FDI) developed for this study.

**Table S3**: Comparison of the LULC map and FDI map. Percentages are based on the LULC map (e.g.: 84.1% intact / intact means that 84.1 of the LULC intact forest pixels belong to the intact forest FDI class).

|  |  | LULC classes | |
| --- | --- | --- | --- |
|  |  | intact | degraded+secondary |
| FDI classes | intact | 84.1 | 56.6 |
|  | light degradation | 10.0 | 14.9 |
|  | moderate to high degradation | 4.6 | 18.0 |
|  | severe degradation | 1.4 | 10.5 |
|  | All degradation levels | 15.9 | 43.4 |

**
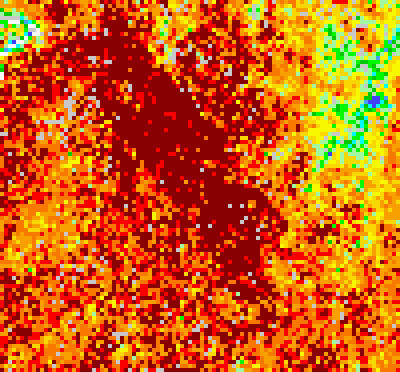
**

**Figure S5**. Example of the effects of RF overfitting. The shape of a Lidar scene is noticeable in the height map.

**SI.7 The leave-one-scene-out as a proxy for the final map validation**

We computed the pixel-level mean difference between the final height map and each of the 47 height maps produced leaving one scene out. We found that the mean difference at pixel level over the whole map was 0.02 ± 0.35m and concluded that we could use these maps to report the uncertainties of the final map for areas covered by lidar.

**Table S4**. Results of the leave-one-scene-out cross validation in relation to TCH, standard deviation of TCH, elevation and standard deviation of elevation from SRTM, sorted by increased TCH.

| scene ID | TCH | stdev TCH | mean elev srtm | stdev elev srtm | R^2^ H map | RMSE H map | bias H map |
| --- | --- | --- | --- | --- | --- | --- | --- |
| 7  37 | 0.83488  2.42792 | 0.78877  3.07756 | 3.7613  10.7585 | 1.44872  2.01733 | 0.63  0.69 | 1.74  1.9 | 0.9  0.8 |
| 9 | 2.99601 | 2.75099 | 5.464 | 2.92108 | 0.41 | 2.59 | -0.25 |
| 38 | 3.17352 | 2.41999 | 7.02802 | 2.6763 | 0.6 | 2.3 | -1.45 |
| 8 | 4.9114 | 3.25462 | 6.42665 | 3.5252 | 0.59 | 3.04 | 2.16 |
| 6 | 9.49934 | 8.78038 | 14.8354 | 10.677 | 0.93 | 2.47 | 0.42 |
| 32 | 11.0672 | 8.05589 | 126.499 | 154.512 | 0.84 | 7.19 | -6 |
| 44 | 12.1651 | 9.48017 | 11.3828 | 7.04095 | 0.81 | 3.99 | -0.62 |
| 46 | 12.3941 | 5.83331 | 11.8494 | 4.65491 | 0.65 | 3.45 | -0.35 |
| 41 | 12.9078 | 3.60709 | 15.3469 | 5.66948 | 0.56 | 3.03 | -1.47 |
| 30 | 12.9887 | 4.97203 | 20.8158 | 7.59394 | 0.46 | 3.97 | 0.05 |
| 28 | 13.1559 | 6.28588 | 14.869 | 6.45076 | 0.63 | 4.14 | 1.45 |
| 45 | 14.1556 | 3.52327 | 12.8539 | 3.05054 | 0.48 | 2.66 | 0.4 |
| 18 | 15.0945 | 5.73464 | 45.9193 | 29.2778 | 0.42 | 4.95 | -1.81 |
| 21 | 15.6501 | 5.84546 | 48.1867 | 20.9116 | 0.54 | 4.48 | -1.55 |
| 27 | 16.3082 | 4.94966 | 36.7876 | 14.7217 | 0.62 | 4.1 | -2.67 |
| 15 | 16.3876 | 5.35047 | 15.128 | 4.67475 | 0.56 | 3.7 | 1.55 |
| 25 | 16.9759 | 5.47228 | 31.3087 | 17.964 | 0.58 | 3.87 | -2.44 |
| 42 | 17.0712 | 6.23583 | 23.9313 | 16.746 | 0.4 | 4.92 | 0.98 |
| 1 | 17.3711 | 1.96526 | 61.0707 | 12.3884 | 0.01 | 5.7 | -5.09 |
| 2 | 17.9886 | 5.50585 | 39.0318 | 17.1178 | 0.48 | 4.42 | -1.81 |
| 13 | 18.3331 | 7.30258 | 26.1322 | 14.2673 | 0.69 | 4.28 | 1.98 |
| 34 | 18.5149 | 7.18795 | 29.1347 | 15.6359 | 0.77 | 3.6 | 0.63 |
| 26 | 18.6642 | 4.73518 | 98.2945 | 31.1943 | 0.28 | 6.35 | -4.86 |
| 23 | 18.8184 | 6.29228 | 49.7748 | 38.2607 | 0.76 | 3.04 | -0.46 |
| 19 | 19.0611 | 5.87961 | 47.9725 | 16.2818 | 0.72 | 3.26 | -0.28 |
| 39 | 20.9855 | 7.02846 | 569.199 | 199.861 | 0.07 | 8.72 | -5.39 |
| 35 | 21.1601 | 4.43562 | 35.7235 | 24.584 | 0.44 | 5.38 | 4.01 |
| 4  0 | 21.1795  21.7417 | 6.589  6.06758 | 44.1218  40.8315 | 30.9046  24.0551 | 0.72  0.59 | 3.87  4.22 | 2.1  2.34 |
| 5 | 21.7667 | 4.39222 | 103.055 | 31.2294 | 0.36 | 4.33 | -2.47 |
| 12 | 21.838 | 4.5282 | 57.3816 | 29.7594 | 0.52 | 3.31 | -0.05 |
| 3 | 22.5529 | 4.84334 | 95.9224 | 26.4161 | 0.23 | 4.89 | -2.35 |
| 20 | 23.1402 | 3.25929 | 74.1515 | 32.4585 | 0.34 | 2.87 | -0.36 |
| 43 | 23.2317 | 6.19841 | 47.3308 | 16.5501 | 0.36 | 4.99 | 0.05 |
| 48 | 23.2889 | 4.38639 | 105.52 | 41.5729 | 0.45 | 3.35 | -0.78 |
| 11 | 23.6028 | 7.27959 | 70.7572 | 43.2771 | 0.74 | 4.17 | 2.49 |
| 17 | 24.0166 | 5.88219 | 82.2057 | 25.9547 | 0.26 | 5.18 | -0.81 |
| 10 | 24.3782 | 3.89221 | 62.6434 | 25.8944 | 0.37 | 3.39 | 1.35 |
| 47 | 24.8083 | 2.34614 | 115.859 | 28.7657 | 0.03 | 2.65 | 0.65 |
| 31 | 25.0606 | 4.38939 | 589.146 | 251.539 | 0.08 | 4.33 | 0.52 |
| 40 | 25.219 | 4.76881 | 682.472 | 276.928 | 0 | 5.28 | 1.95 |
| 14 | 25.952 | 4.97616 | 63.1412 | 20.5435 | 0.54 | 5.33 | 4.1 |
| 16 | 26.3432 | 2.62867 | 127.02 | 27.6471 | 0.05 | 3.74 | 1.24 |
| 33 | 27.0128 | 4.58143 | 307.226 | 157.982 | 0.11 | 4.89 | 2.24 |
| 24 | 28.8161 | 3.84411 | 126.767 | 106.65 | 0.19 | 6.97 | 5.93 |

**Figure S6.** Semivariograms of the Height uncertainty (left) and AGB uncertainty (right). A piecewise exponential model was used to fit the data.

**Figure S7.** Measure of importance for each predictor variable. For any variable, the measure is the increase in prediction error if the values of that variable are permuted across the out-of-bag observations. This measure is computed for every tree, then averaged over the entire ensemble and divided by the standard deviation over the entire ensemble (<https://www.mathworks.com>).

**References**

Dijkshoorn K, Huting J, Tempel P. Update of the 1: 5 million soil and terrain database for Latin America and the Caribbean (SOTERLAC). ISRIC Rep. 2005 Jul;1(1):25.

Duque A, Saldarriaga J, Meyer V, Saatchi S. Structure and allometry in tropical forests of Chocó, Colombia. Forest Ecology and Management. 2017 Dec 1;405:309-18.

Exelis VI. IDL software.

Hijmans RJ, Cameron S, Parra J. WorldClim climate surfaces. Online at http://biogeo. berkeley. edu/worldclim/worldclim. htm. 2004.

https://www.asf.alaska.edu/sar-data/palsar/ [October 27^th^, 2018]

<https://earthengine.google.com> [October 27^th^, 2018]

https://www.mathworks.com [October 27^th^, 2018]

https://www.climatelinks.org/resources/bioredd-colombia [Oct 27^th^ 2018]

<https://www2.jpl.nasa.gov/srtm/> [October 27^th^, 2018]

Malhi Y, Roberts JT, Betts RA, Killeen TJ, Li W, Nobre CA. Climate change, deforestation, and the fate of the Amazon. science. 2008 Jan 11;319(5860):169-72.

Meyer V, Saatchi S, Clark DB, Keller M, Vincent G, Ferraz A, Espírito-Santo F, d'Oliveira MVN, Kaki D, Chave J. Canopy area of large trees explains aboveground biomass variations across neotropical forest landscapes. Biogeosciences. 2018;15, 3377-3390, <https://doi.org/10.5194/bg-15-3377-2018>.

Rodríguez-Veiga P, Saatchi S, Tansey K, Balzter H. Magnitude, spatial distribution and uncertainty of forest biomass stocks in Mexico. Remote Sensing of Environment. 2016 Sep 15;183:265-81.

Saatchi SS, Harris NL, Brown S, Lefsky M, Mitchard ET, Salas W, Zutta BR, Buermann W, Lewis SL, Hagen S, Petrova S. Benchmark map of forest carbon stocks in tropical regions across three continents. Proceedings of the national academy of sciences. 2011 Jun 14;108(24):9899-904.

Shimada M, Isoguchi O, Tadono T, Isono K. PALSAR radiometric and geometric calibration. IEEE Transactions on Geoscience and Remote Sensing. 2009 Dec;47(12):3915-32.

Sombroek W. Amazon landforms and soils in relation to biological diversity. Acta Amazonica. 2000 Mar;30(1):81-.

Xu L, Saatchi SS, Yang Y, Yu Y, White L. Performance of non-parametric algorithms for spatial mapping of tropical forest structure. Carbon balance and management. 2016 Dec;11(1):18.
